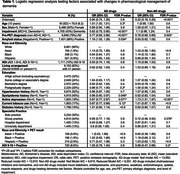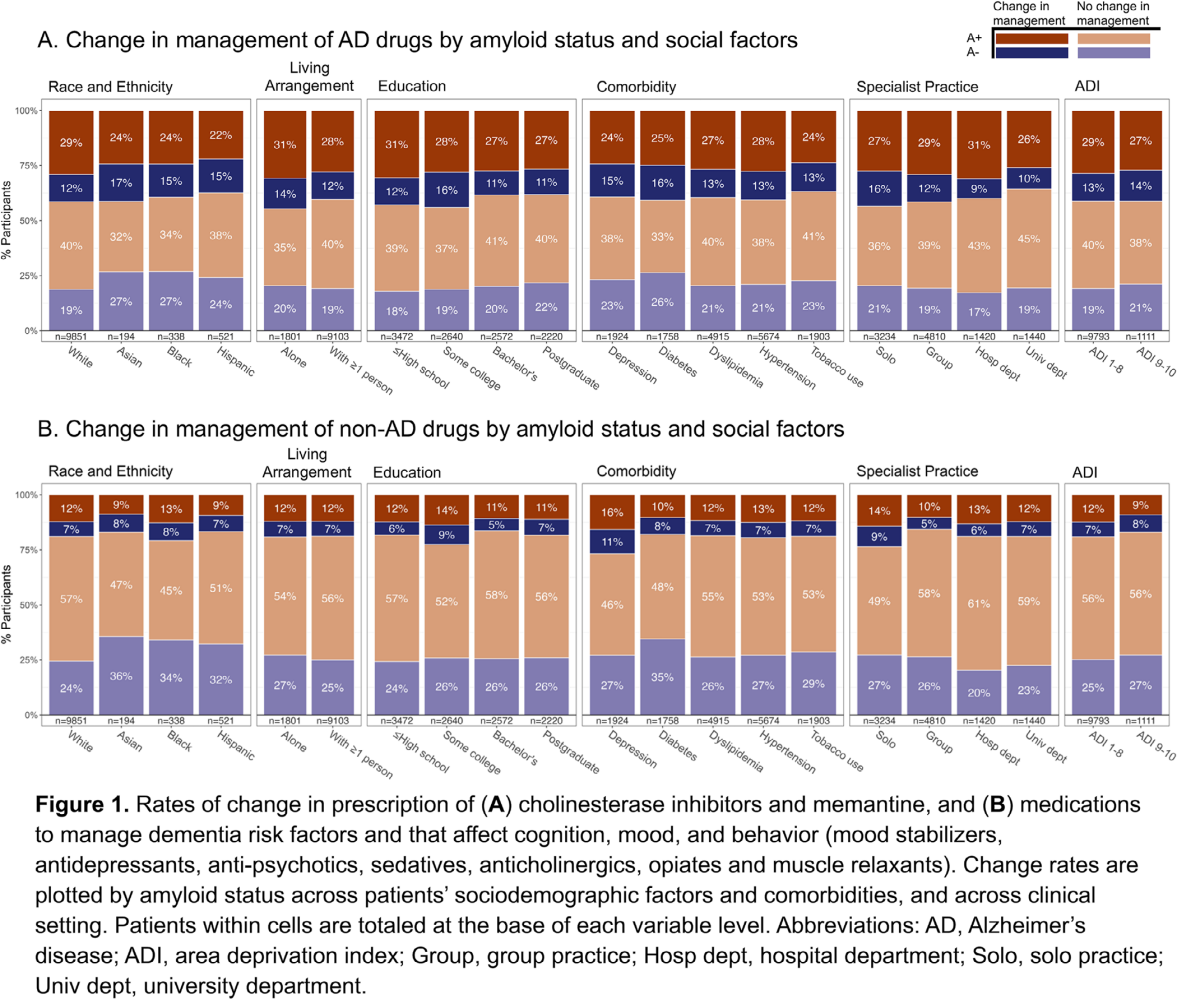# Social determinants of pharmacological dementia management: results from IDEAS

**DOI:** 10.1002/alz70862_110237

**Published:** 2025-12-23

**Authors:** Margo B. Heston, Charles C. Windon, Lucy Hanna, Constantine Gatsonis, Maria C. Carrillo, Charles Apgar, Peggye Dilworth‐Anderson, Bruce E Hillner, Andrew March, Barry A Siegel, Rachel A. Whitmer, Consuelo H. Wilkins, Renaud La Joie, Gil D. Rabinovici

**Affiliations:** ^1^ University of California, San Francisco, San Francisco, CA USA; ^2^ Dept. of Biostatistics, Brown University, Providence, RI USA; ^3^ Alzheimer's Association, Chicago, IL USA; ^4^ American College of Radiology, Reston, VA USA; ^5^ University of North Carolina, Chapel Hill, Chapel Hill, NC USA; ^6^ Virginia Commonwealth University, Richmond, VA USA; ^7^ Washington University in St Louis, St. Louis, MO USA; ^8^ University of California, Davis, Davis, CA USA; ^9^ Vanderbilt University Medical Center, Nashville, TN USA

## Abstract

**Background:**

Socioeconomic vulnerabilities and healthcare environment contribute to disparities in dementia assessment. Whether these affect dementia management, however, remains unclear. We used Imaging Dementia – Evidence for Amyloid Scanning (IDEAS) study data to compare the impact of amyloid PET on pharmacological management across social factors, patient comorbidities, and physician practice settings.

**Methods:**

We analyzed rates of pharmacological change in IDEAS participants with visually interpretable amyloid PET, completed pre‐ and post‐PET case reports, social determinants (racioethnic identity of Asian, Black, Hispanic, or White, area deprivation index (ADI), living arrangement, education) and medical history. Outcomes included any change between pre‐PET and post‐PET visits in prescription of Alzheimer’s disease (AD) drugs, and of non‐AD drugs treating dementia risk factors or affecting cognition/mood/behavior. We used multilevel logistic regression with a random site intercept to test whether the probability of change in management associated with social determinants, race/ADI interactions with amyloid‐positivity, comorbidities, and clinical setting, adjusting for demographics.

**Results:**

Among 10,904 cognitively impaired participants (Table 1), 90% were White, with 4.8% Hispanic, 3.1% Black, and 1.8% Asian representation. 10% resided in highly disadvantaged neighborhoods (ADI 9‐10), 83% lived with ≥1 person, and 68% were educated past high school.

Pre‐FDR correction (adjusted *P*‐values: Table 1), AD drug management change was associated with dyslipidemia (OR [95% CI]=0.88 [0.80‐0.97], *P*_unadj_=.007), depression (0.87 [0.77, 0.97], *P*_unadj_=.014), and tobacco use (0.87 [0.77, 0.99], *P*_unadj_=.028). Non‐AD drug management change was associated with depression (1.73 [1.51, 1.97], *P*_unadj_<.001), group practice (0.72 [0.56, 0.93], *P*_unadj_=.012) and ADI in amyloid‐positive participants (0.68 [0.47, 0.98], *P*_unadj_=.04). Change rates were also associated with amyloid‐PET status, impairment level, and etiology. Figure 1 summarizes change rates across social factors.

**Conclusions:**

These results suggest amyloid status, cognitive impairment level, dementia etiology, and comorbidities may inform pharmacological decision‐making. Clarifying dementia etiology with amyloid PET may, for instance, help clinicians optimize treatment plans to address undermanaged depression in cognitively impaired older adults. Further, socioeconomic disadvantage may limit clinical response to amyloid‐positivity. Replication in New IDEAS and examination of Medicare claims will help elucidate whether disparities in pre‐PET management and management changes are primarily driven by access barriers to assessment and healthcare.